# Survival strategies of an anoxic microbial ecosystem in Lake Untersee, a potential analog for Enceladus

**DOI:** 10.1038/s41598-022-10876-8

**Published:** 2022-05-05

**Authors:** Nicole Yasmin Wagner, Dale T. Andersen, Aria S. Hahn, Sarah Stewart Johnson

**Affiliations:** 1 Department of Biology, Georgetown University, 3700 O Street NW, Washington, DC 20057 USA; 2 SETI Institute, Mountain View, CA USA; 3 Koonkie Inc, Menlo Park, CA USA; 4 Science, Technology, and International Affairs Program, Georgetown University, Washington, DC USA

**Keywords:** Astrobiology, Ecosystem ecology, Limnology

## Abstract

Lake Untersee located in Eastern Antarctica, is a perennially ice-covered lake. At the bottom of its southern basin lies 20 m of anoxic, methane rich, stratified water, making it a good analog for Enceladus, a moon of Saturn. Here we present the first metagenomic study of this basin and detail the community composition and functional potential of the microbial communities at 92 m, 99 m depths and within the anoxic sediment. A diverse and well-populated microbial community was found, presenting the potential for Enceladus to have a diverse and abundant community. We also explored methanogenesis, sulfur metabolism, and nitrogen metabolism, given the potential presence of these compounds on Enceladus. We found an abundance of these pathways offering a variety of metabolic strategies. Additionally, the extreme conditions of the anoxic basin make it optimal for testing spaceflight technology and life detection methods for future Enceladus exploration.

## Introduction

### Antarctic Lake Untersee

The discovery of extreme environments and the study of the organisms that inhabit them have made the search for life beyond Earth a more plausible endeavor^1^. One of the most extreme environments on Earth is Antarctica, a vast polar desert where temperatures dip to the lowest levels on the planet. Where local conditions allow for the presence of liquid water, isolated oases of life exist^2^. These include ice-covered lakes where microbial extremophiles have developed strategies to live in cold, low-light, oligotrophic conditions. While seasonally ice-covered lakes tend to have geochemically different environments in winter and summer, perennially ice-covered lakes in Antarctica are more physiochemically stable^3,4^. The thick, perennial ice-cover dampens the effects of turbulent winds and attenuates the penetration of summer sunlight, which then drops to zero during the long polar winter.

One such lake, Untersee, is a perennially ice-covered, ultra-oligotrophic lake located at 71.34°S, 13.46°E in the Gruber Mountains of central Dronning Maud Land^5–7^. Elevations in the oasis range from 600 to 2790 m and local geology consists of norite, anorthosite, and anorthosite–norite alternations of the Eliseev anorthosite massif^8^. The lake is located within a closed basin at c. 610 m a.s.l. and is dammed at its northern end by the Anuchin Glacier where pressure ridges form at the lake–glacier interface. Lake Untersee is 2.5 km wide and 6.5 km long and is among the largest lakes in central Queen Maud Land^5^.

A result of the intense evaporation and sublimation is that summer melt does not provide significant amounts of water for recharge. The only large contributions of liquid water occur from melt of the Anuchin Glacier beneath the lake ice cover, and sub-glacial/groundwater discharge. A 16S rRNA amplicon study showed that this glacier does not function as a standalone ecosystem but functions as part of the lake itself as a larger scale microbial system^52^. The lack of streams entering into the lake or formation of summer moats results in an environment that is essentially sealed off, with no direct contact with the atmosphere^5,9^.

**Figure 1:**
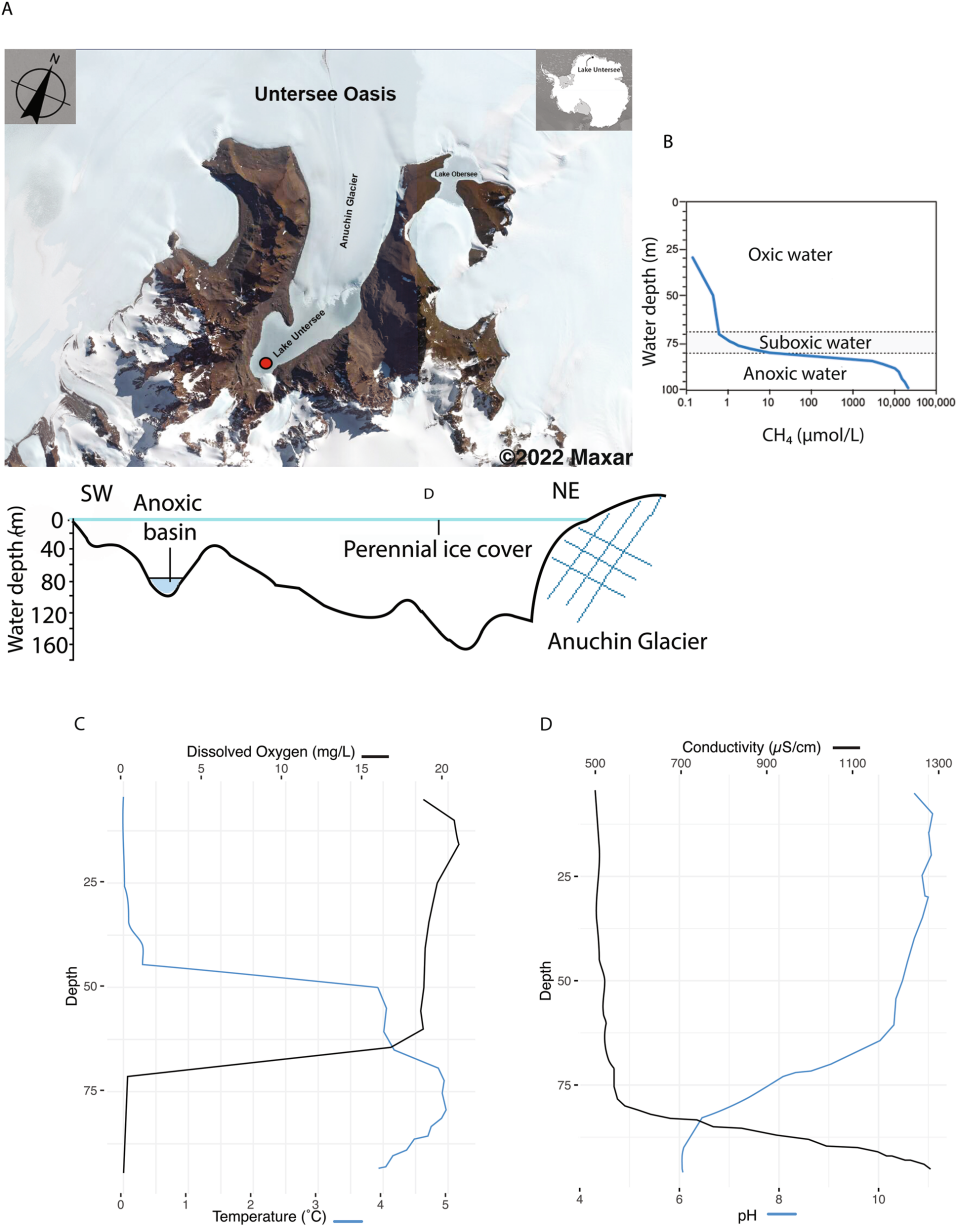
An Overview of Lake Untersee’s Anoxic Basin. (**A**) Satellite view of Lake Untersee and a cross-section of the bathymetry of the lake with the location of the anoxic trough in the South basin. (**B**) Dissolved methane concentrations within the South basin. (**C**) Dissolved oxygen concentrations and temperature within the South basin. (**D**) pH and conductivity in the South basin. Image credit: satellite imagery (©Maxar) provided by NextView License, depth profiles modified from Wand et al., 2006 and Dale Andersen, unpublished data.

Lake Untersee loses c. 1% of its water annually from the sublimation of the 2.5–4 m thick ice cover. To maintain hydrological balance the lake must be recharged by an equal inflow^5,10^. The lake is dammed at its northern end by the Anuchin Glacier and mass balance calculations suggest that subaqueous melting of terminus ice contributes 40–45% of the annual water budget with subglacial meltwater contributing the remainder^5^. Based on δD-δ18O of the water column, the lake has not developed a moat for at least the past 300–500 years providing another indication of just how isolated this lake is from the atmosphere or other external inputs^11^. Information on the properties of the water column can be found in Fig. 1 as well as Supplementary Table S4.

An exclusively microbial ecosystem is found along Lake Untersee’s bed with photosynthetic microbial mat communities observed to depths of at least 160 m^12^ (Dale Andersen, unpublished data). The microbial mats are composed of filamentous cyanophytes that form flat, prostrate mats, cm-scale cuspate pinnacles, and large conical stromatolites that rise up to 70 cm above the lake floor. These larger microbial structures provide an example of modern, unlithified stromatolites with a morphology similar to the large, conical stromatolites that existed during the Archaean era^53^. Two unusual features of the lake are a high concentration of dissolved methane (> 20 mM) in the deep part of the anoxic basin, and a pH of ≥ 10.5 in the mixed layer of the lake. The primary source of methane has been identified as microbial reduction of carbon dioxide using hydrogen^13^. The high pH of the poorly buffered lake water is partly, if not primarily, due to CO_2_ uptake by the photosynthetic microbial mats and subglacial weathering of plagioclase^9,12,14^.

### Possibility of life on Enceladus

**Figure 2:**
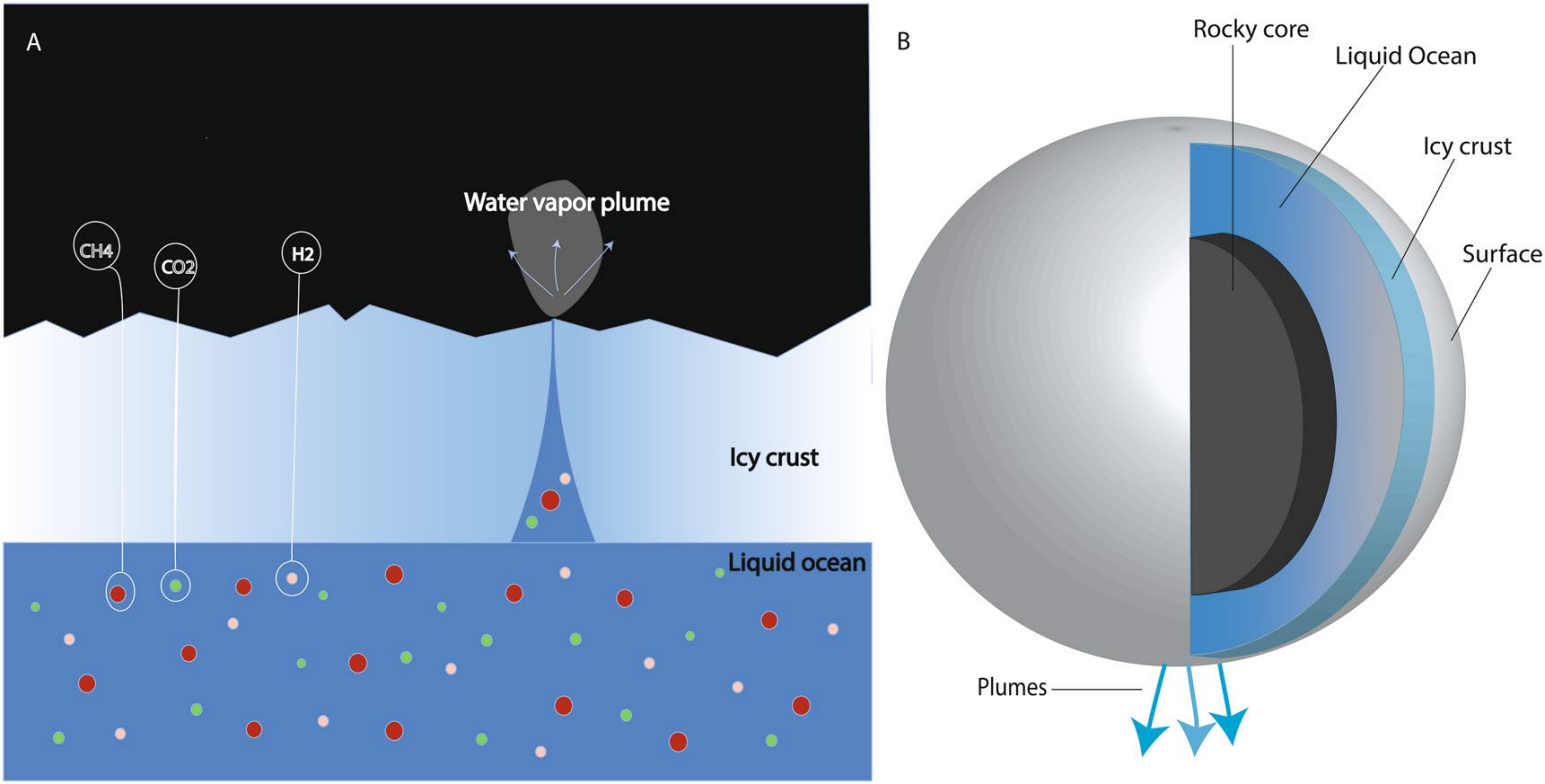
(**A**) View of a cross section of Enceladus and the ocean of Enceladus. (**A**) Enceladus ocean and icy crust. (**B**) Cross section of Enceladus’s icy crust, ocean and core. Image credit: illustration by N. Y. Wagner, drawing upon schematic renderings from NASA/JPL-Caltech and the Southwest Research Institute.

The anoxia, darkness, and chemical composition of Lake Untersee’s anoxic southern basin (dissolved H_2_, CO_2_, CH_4_, NH_3_, etc.) make it a relevant environment for studying the potential for life in the oceans of icy moons in our solar system (Fig. 2)^14^. One of these bodies is Enceladus, the sixth-largest moon of Saturn. Although 25 times smaller than Earth, Enceladus harbors what is believed to be a global ocean covered by a global ice sheet^15^. In 2005, the National Aeronautics and Space Administration’s (NASA) Cassini mission discovered plumes of gas and icy particles venting into space over the Saturnian moon’s south polar region^16,17^. To date, the Cosmic Dust Analyzer (CDA) instrument detected biologically available nitrogen in the form of amines, traces of H_2_S, inorganic salts, and carbon dioxide and molecular hydrogen that can function as a redox pair for methanogenesis in anoxic environments. Methanogenesis is one of the oldest known energetic pathways on Earth. The presence of salts and all the compounds necessary for life (C, H, N, O, P, S) together with the presence of liquid water, indicate a habitable environment^18,19,31^. Future missions searching for evidence of life may collect venting particles during flybys through the plumes, or landers may obtain larger sample quantities from particles that fall back onto the surface of Enceladus, thus providing a means for high-sensitivity measurements for the detection of trace elements and any potential biosignatures.

### Analog study objectives

The deep waters of the southern basin of Lake Untersee harbor carbon dioxide, hydrogen, methane, and ammonia, which in combination with low mean temperatures represents a physical, chemical, and possibly biological analog for Enceladus^9^. Lake Untersee’s anoxic basin dominated by a low-temperature anaerobic microbial ecosystem. Thus studying Lake Untersee’s anoxic basin, especially the energetic pathways within the microbial communities inhabiting the lake, can shed light on the potential habitability of Enceladus. Here, four samples from the anoxic water column as well as a sample of the anoxic sediment were collected. We sequenced the microbial communities using a whole genome shotgun approach in order to chart the taxonomic composition of these communities and identify metabolic pathways and strategies used to survive these extreme conditions.

## Results

Water samples were filtered twice (see Methods), first through a large filter (0.45 µm, LF or “Large Filter”) and then the filtrate was passed through a small filter (0.05 µm, UF or “Ultrafine Fraction”). Using whole genome shotgun metagenomics from four water samples (LF92 and UF92 from the 92 m depth, LF99 and UF99 from the 99 m depth) as well as one sediment sample, we provide the first comprehensive whole genome shotgun metagenomics investigation of this section of the lake and highlight both the taxonomic composition and potential metabolic strategies for survival, as well as identify areas for deeper investigation.

### Cell counts and dissolved nutrients

In order to determine the habitability of the anoxic basin, the cell counts were measured in the oxycline (75 m depth) and the anoxic region (92 and 99 m depth), where oxygen content is < 1 mg/L (Fig. 1). The cell counts were 84,129 cells/mL at 75 m, 895,516 cells/mL at 92 m, and 775,404 cells/mL at 99 m. In the anoxic depths, the cell counts were roughly an order of magnitude higher than the count at 75 m depth. Dissolved nutrient levels were also significantly higher in the anoxic section than the oxycline (Table 1). The ammonium content at 92 m was 1675.98 µmol/L, double the amount at 99 m, which was 758.53 µmol/L. The concentration was much lower in the oxycline at 4.11 µmol/L. The phosphate content in the oxycline was 0.06 µmol/L. The concentration was over three orders of magnitude higher in the anoxic region, measuring 35.19 µmol/L at the 92 m depth and 47.30 µmol/L at the 99 m depth. The silicate content in the oxycline was 86.76 µmol/L. The concentration was once again higher in the anoxic region, measuring 387.62 µmol/L at 92 m and 664.60 µmol/L at 99 m.

**Table 1:** Summary of anoxic lake properties, including cell counts and dissolved nutrients.

Site	Cell count #cells/mL	Nitrate + Nitrite µmol/L	Ammonium µmol/L	Phosphate µmol/L	Silicate µmol/L
75 m	84,129	< 0.040	4.11	0.06	86.76
92 m	895,516	< 0.040	1675.98	35.19	387.62
99 m	775,404	< 0.040	758.53	47.30	664.60

### Taxonomic profiling

#### Unclassified organisms

Taxonomic annotations were performed using the Lowest Common Ancestor Star algorithm (LCA*) with the RefSeq database^21^. With this annotation tool, any organism that could not be assigned a taxonomic classification beyond the superphylum level (bacteria, archaea, prokaryotes, root) was designated as “unclassified”. LCA* was able to classify an average of 60% of the contigs across all samples. Kaiju^22^ and Kraken2^23^ were able to classify an average of 18.75% and 19.5% of the reads from all samples respectively. Given that LCA* was able to annotate the greatest fraction of the reads, we focused on these results. The percentage of unclassified organisms increased with depth, from 37% at 92 m to 43% at 99 m to 45% of the community in the sediment layer. At the phylum level, 97–99% of the unclassified organisms were assigned to the bacterial superphylum. In each sample, fewer than three percent of the unclassified organisms belonged to the archaea superphylum (Figure S1).

#### Classified organisms

Archaea make up 2% of classified organisms at the 92 m depth and 4% of classified organisms at the 99 m depth and in the sediments. Bacteria make up 98% of classified organisms at the 92 m depth, and 96% of classified organisms at 99 m depth and in the sediment. Specifics of classified and unclassified organisms per sample as well as deeper taxonomic classifications may be found in the Supplementary Information (Figures S1 and S2).

##### Classified archaea

**Figure 3:**
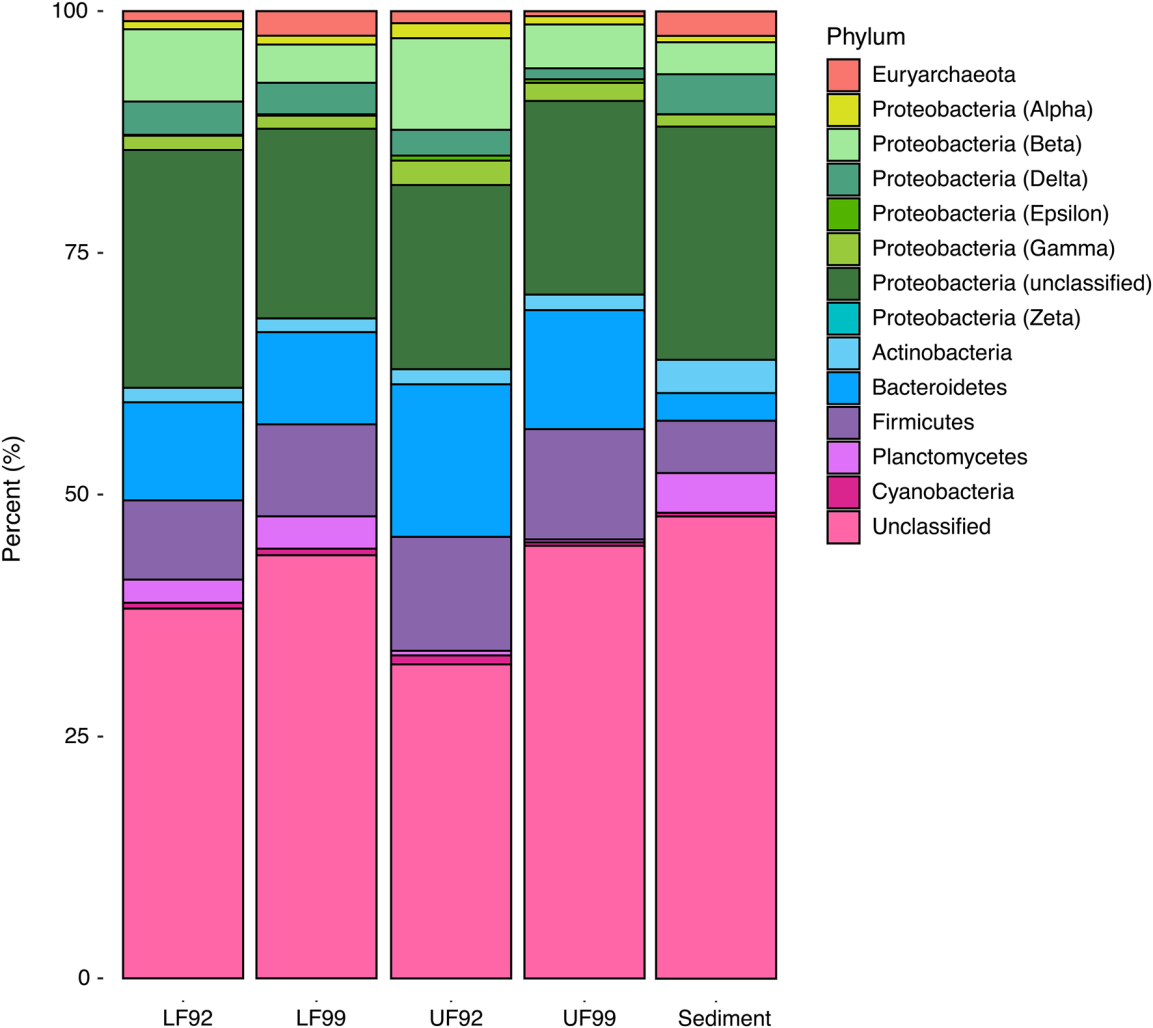
The taxonomic composition of the communities at the phyla level. Classified organisms in the figure only include phyla that make up more than 1% of the community.

The total percentage of archaea in the samples doubles from 1% of the community at the 92 m depth to 2% at 99 m and in the sediment (Fig. 3). Archaea are almost entirely classified at or beyond the phylum level in these samples. Euryarchaea are the most abundant archaeal phylum in all samples. They make up 97% of archaea, or 1% of all organisms at the 92 m depth. At the 99 m depth and the sediment layer, they make up over 99% of archaea, or 2% of all organisms. Euryarchaea are well-known anoxic methanogens, and their higher abundance at 99 m and in the sediment could explain why the concentration of dissolved methane was found to be highest at the deep anoxic layer near the water–sediment interface (Fig. 1)^13^. More details on the archaeal community may be found in Table 2 and in the Supplementary Information.

**Table 2:** Summary of organisms in the anoxic basin. Taxonomic abundances in different samples are based on results from LCA* (RefSeq database).

			LF92 (%)	UF92 (%)	UF99(%)	LF99 (%)	Sediment (%)
Unclassified Organisms			36	30	42	41	45
Bacteria	Alphaproteobacteria	Rhizobiales	1	2	1	1	1
		Rhodospirillales					
		other					
	Betaproteobacteria	Burkholderiales	8	10	5	4	6
		Other					
	Deltaproteobacteria	Desulfovibrionales	16	9	9	13	15
		Myxococcales					
		Desulfobacterales					
		Dusulfarculaceae					
		Desulfuromonadales					
		Other					
	Gammaproteobacteria	Pseudomonadale	2	3	2	2	1
		Mythlococcales					
		Other					
	Bacteroidetes	Marinifilaceae	10	15	12	9	3
		Cytophagaceae					
		Chitinophagaceae					
		Other					
	Firmicutes	Clostridia	8	11	11	9	5
		Bacilli					
		Other					
	Planctomycetes	Planctomycetia	2	0.50	0.30	3	4
		Other					
	Actinobacteria	Actinobacteria	1	1	2	1	3
		other					
	Other	Other	25	27	25	25	25
Archaea	Thermoprotei	Other	0.02	0.01	0	0.02	0.002
	Thaumarchaea	Other	0.005	0.007	0.000	0.010	0.002
	Euryarchaea	Methanomicrobia	1	1	0.50	2	2
		Methanobacteriales					
		Methanococcales					
		Other					
	Other		0.001	0.001	0.000	0.006	0.008

##### Classified bacteria

Proteobacteria are the most abundant classified bacteria in the system. The classes present include Alphaproteobacteria, which make up 1% of organisms in LF92, 2% in UF92, and 1% in LF99, UF99, and the sediment. Betaproteobacteria make up 8% of organisms in LF92, 10% of organisms in UF92, 5% in UF99, 4% in LF99, and 6% in the sediment. Deltaproteobacteria make up 16% of the community in LF92, 9% in UF92 and UF99, 13% in LF99, and 15% in the sediment. Gammaproteobacteria make up 2% of the organisms in LF92, 3% of organisms in UF92, 2% in UF99 and LF99, and 1% of the community in the sediment. Planctomycetes make up 2% of all organisms in LF92, 0.5% of organisms in UF92 and 0.3% in UF99, and 3% of organisms in LF99. The most abundant class belonging to this phylum is Planctomycetia. Actinobacteria make up 1% of organisms in the LF92, UF92 and LF99 samples, 2% of organisms in UF99, and a total of 3% of classified organisms. The only major annotated class within Actinobacteria is also named Actinobacteria and makes up more than 70% of this phylum in the water column and 48% of the phylum in the sediment. Bacteroidetes make up 10% of the community in LF92, 15% of the community in UF92, 12% of the community in UF99, 9% of the community in LF99 and 3% of the community in the sediment. The major annotated classes belonging to Bacteroidetes are Marinifilaceae, Cytophagaceae and Chitinophagaceae. Firmicutes constitute 8% of the organisms within LF92, 11% of UF92 and UF99, 9% of LF99, and 5% of the sediment. The major annotated classes within this phylum are Clostridia and Bacili. A summary can be found in Table 2 and more detail is included in the Supplementary Information.

### Taxonomic differences between fraction sizes

To ascertain if cell size played a role in survival capabilities in this extreme environment, we assessed the metabolic potential within the size fractions. While the percentage of Proteobacteria in the community is more related to depth than filter size in the water column, within the Proteobacteria, we found the large fraction samples had 13–16% of the community classified as Deltaproteobacteria. In contrast, only 9% belong to ultrafine fractions. At 15%, the fraction of Deltaproteobacteria in the sediment is similar to that of the LF samples. Planctomycetes make up 2–3% of the LF samples, while they make up only 0.3–0.5% of the UF communities. Planctomycetes make up 4% of the community within the sediment. More details on these differences can be found in Table 2. The size of the organisms did not seem to affect survival.

### Functional analysis and metabolic pathways of interest

To determine how organisms use energy sources available to them, MetaPathways V2.5^24^ was used to assign metabolic pathways to the open reading frames (ORFs) and predicted metabolic pathways using pathwaytools^25^. An average of 830 (standard deviation of 170) pathways per sample were identified. 310 pathways were shared among all samples while an average of 27 (standard deviation of 15) pathways were unique to each sample. An average of 43 pathways were unique to unclassified organisms among the samples, and 101 pathways were unique to classified organisms among all samples**.** Overall, an average of 1.3% of all the pathways were responsible for biosynthesis, 14.2% of pathways were responsible for degradation, and an average of 0.2% of all the pathways belonged to metabolite precursor generation. The greatest pathways abundance, with an average of 84.1% of all pathways, belonged to energy metabolism. To link function and taxonomy, we used the LCA annotation (obtained using the RefSeq database^26^) assigned to the ORF that corresponded to a pathway.

Next, we focused on pathways relevant to the survival on Enceladus. First, the metabolic pathways related to methanogenesis were examined; we then investigated pathways related to nitrogen and sulfur compounds that have either been detected or could potentially exist on Enceladus.

#### Methane metabolic pathways

**Figure 4:**
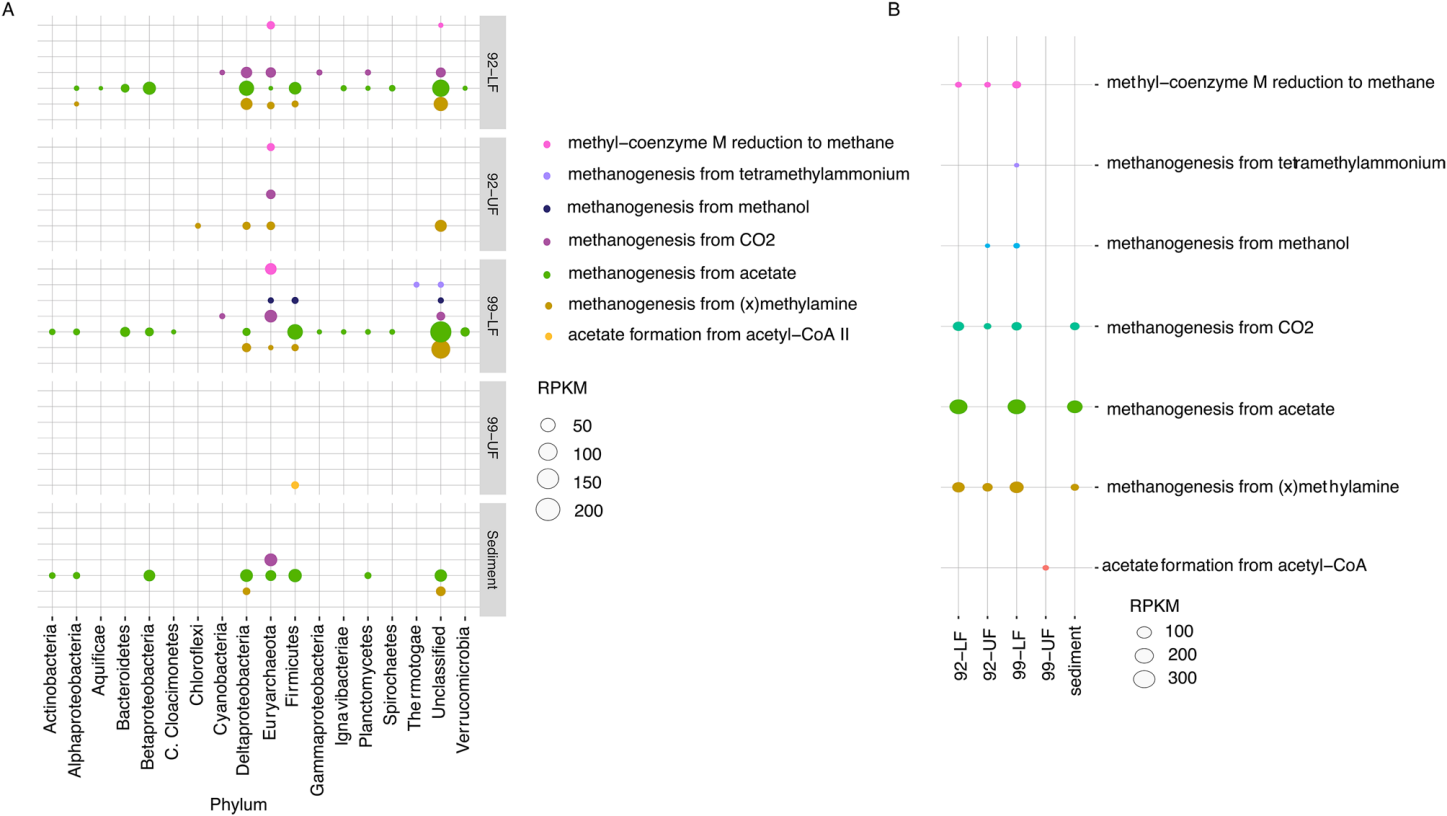
An overview of the methanogenesis pathways. (**A**) Shows the methanogenesis pathway abundances in the phyla. (**B**) Indicates the methanogenesis pathway abundances in each sample. Since only 44% of the reads mapped back to the UF99 sample, not much information can be inferred from the data belonging to this sample. Here, we view methane metabolism from carbon dioxide to be the main pathway present in Euryarchaea (the only known anoxic methanogens). Methanogenesis from acetate is another pathway seen abundantly in non-methanogens. This could indicate acetate as a source of energy in this anoxic environment.

Because the high concentration of dissolved methane in the anoxic water is believed to be due to biotic methanogenesis^13^, the pathways for methanogenesis in the environment were explored and linked to the organisms annotated to those pathways (Fig. 4). Methanogenesis from (x)methylamine resulted in three times fewer methanogenesis pathways in LF92 (21.2%) and LF99 (26.40%) than in UF92 (70.40%). In the sediments, however, it made up only 9% of methanogenesis pathways. In the water column, these pathways were present in unclassified organisms, Firmicutes, Deltaproteobacteria, and Euryarchaea. In the sediments, (x)methylamine methanogenesis pathways were only present in unclassified organisms and Deltaproteobacteria.

Methanogenesis from CO_2_ was most abundant in the UF92 (19.8%) and LF92 (16.3%) samples, at almost double what was found in LF99 (9.7%). In the sediments, 16% of methanogenesis pathways belonged to methanogenesis from CO_2_. Methanogenesis from CO_2_ was the main methanogenesis pathways present in Euryarchaea in every sample. Except for LF92, pathways for methanogenesis from CO_2_ were only present in Euryarchaea and unclassified organisms.

Pathways belonging to methanogenesis from acetate were similar among LF92 (60.5%), LF99 (54.9%), and the sediment (74.9%). In all samples, methanogenesis from acetate was the most abundant in unclassified organisms, Deltaproteobacteria, Betaproteobacteria, and Firmicutes.

#### Nitrogen

**Figure 5:**
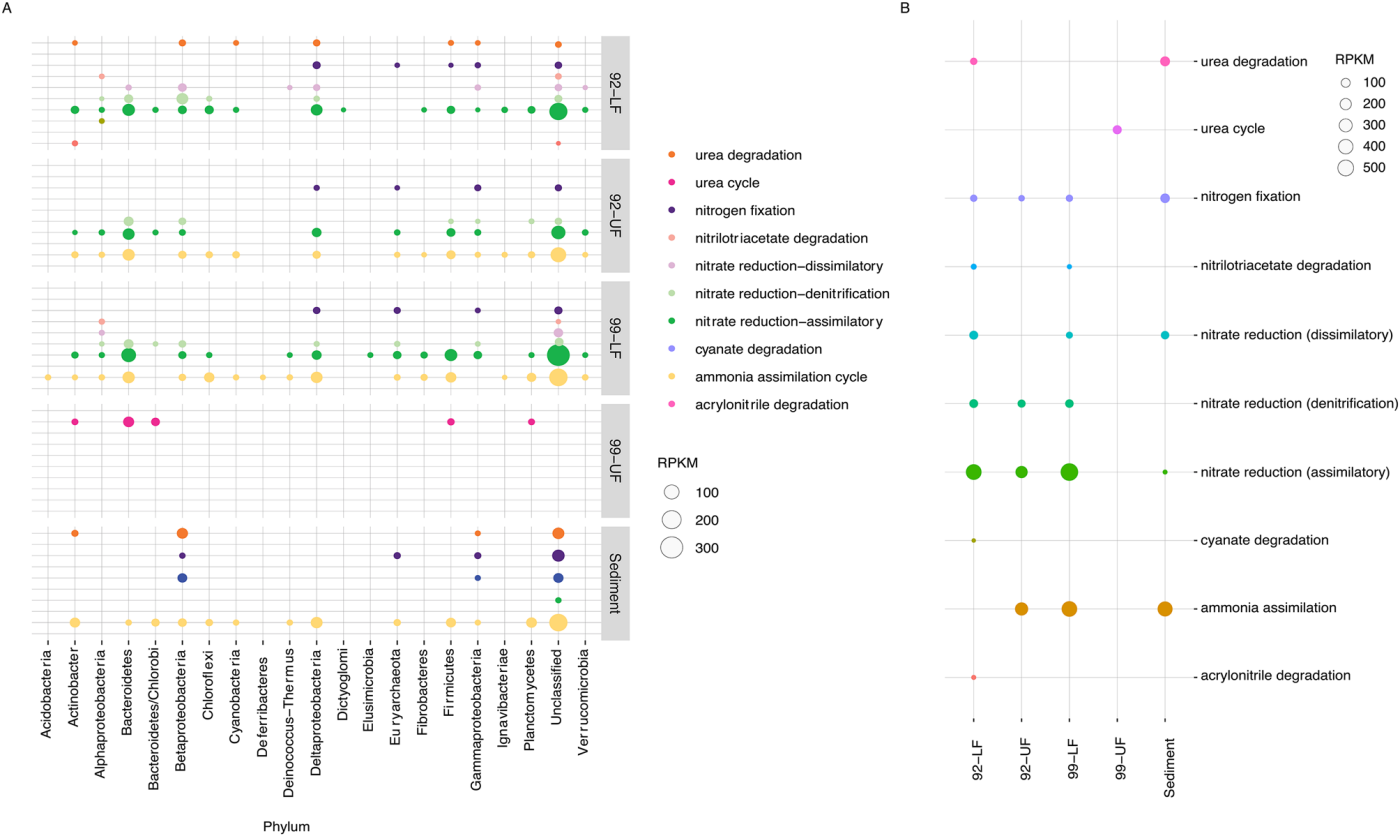
An overview of the nitrogen metabolism pathways. (**A**) Shows the nitrogen metabolism pathway abundances in the phyla. (**B**) Shows the nitrogen metabolism pathways abundance in each sample. Since only 44% of the reads mapped back to the UF99 sample, not much information can be inferred from the data belonging to this sample. Nitrate reduction, the most abundant nitrogen metabolism reaction, can couple with methane oxidation and facilitate the usage of methane as an energy source.

Nitrogen metabolism plays an important role in every ecosystem and nitrogen-bearing organic compounds have been detected in the plumes of Enceladus. In order to determine how nitrogen is cycled in this environment, we investigated the presence and abundance of nitrogen metabolic pathways and found that nitrate reduction pathways were the most abundant in our samples, including assimilatory, dissimilatory, and denitrification pathways. While assimilatory and denitrification pathways were mainly present in the water column (and were the most abundant in the water), other nitrogen pathways were present in both water and within the sediment, including assimilatory nitrate reduction, nitrogen fixation, ammonia assimilation, urea degradation, and denitrification (Fig. 5).

Assimilatory nitrate reduction was the most abundant nitrogen pathway, making up between 39–64.9% of nitrogen metabolism pathways in the water column and only 4.7% within the sediment community (Fig. 5). This pathway was mainly found in unclassified organisms, but was also present in large abundance in Deltaproteobacteria, Bacteroidetes, and Firmicutes.

The ammonia assimilation cycle made up between 35.4 and 49.2% of nitrogen metabolism pathways in the water column, however, unlike assimilatory nitrate reduction, in the sediment this pathway represented 60.4% of the nitrogen metabolism pathways. This pathway was present in nearly all major phyla in UF92, LF99, and the sediment. It was most abundant in unclassified organisms.

Denitrification was present in unclassified organisms and Bacteroidetes. This pathway made up 9% of nitrogen metabolism pathways in the water column but was absent in the sediment, suggesting that the environmental conditions in the sediment are not conducive to denitrification.

#### Sulfur

**Figure 6:**
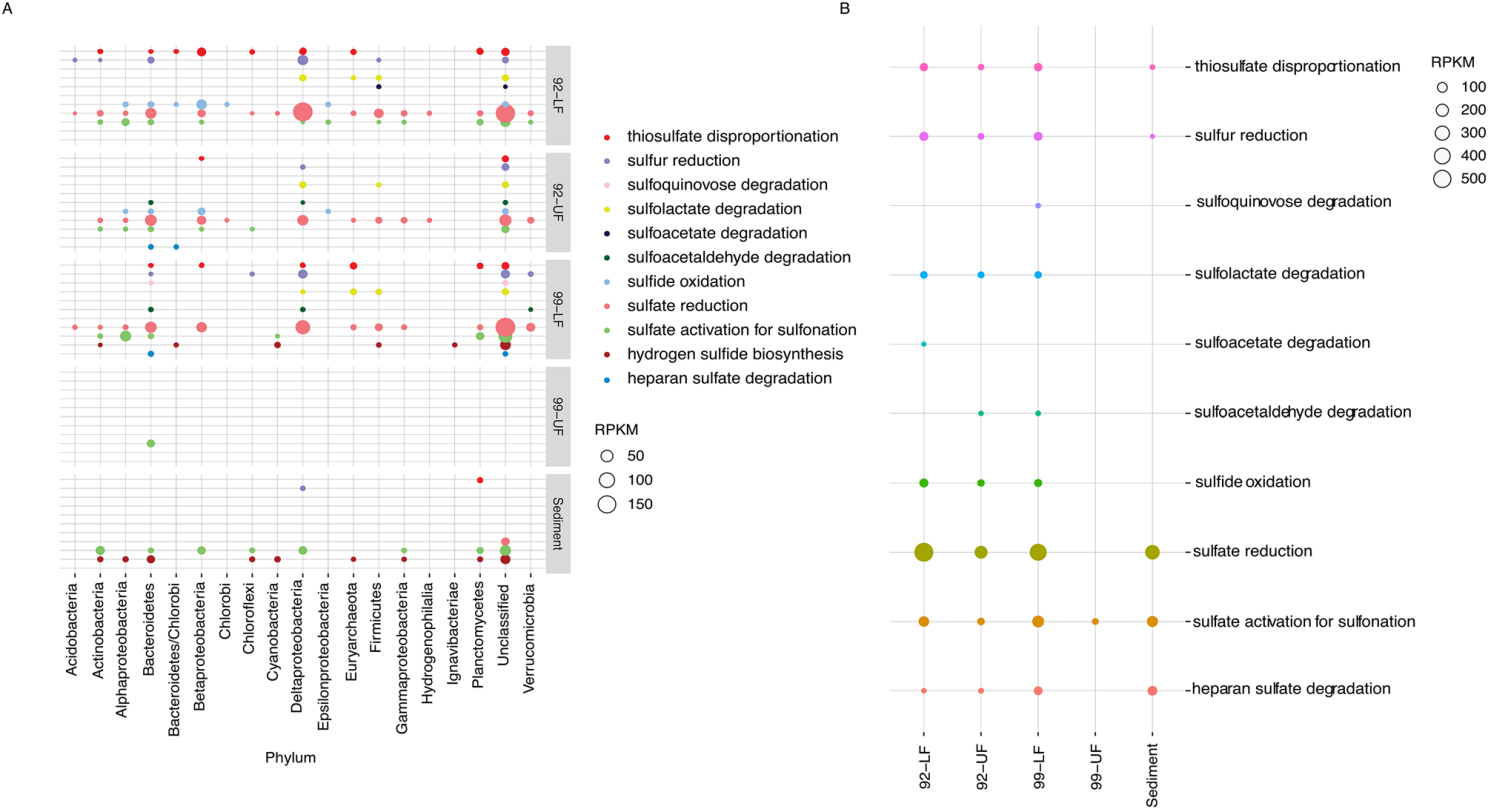
An overview of the sulfur metabolism pathways. (**A**) Shows the sulfur metabolism pathway abundances in the phyla. (**B**) Shows the sulfur metabolism pathways abundance in each sample. Since only 44% of the reads mapped back to the UF99 sample, not much information can be inferred from the data belonging to this sample. Sulfate reduction, the most abundant sulfur metabolism reaction, can couple with methane oxidation and facilitate the usage of methane as an energy source.

Sulfate is an effective electron acceptor in anoxic environments and an important metabolic compound in anoxic environments. Additionally, current evidence suggests that a possible biosphere within the oceans of Enceladus may not be limited by the availability of sulfur^31^. Sulfur reducing bacteria and methane oxidizers have been found to work alongside each other. Given the connection of sulfur pathways with methane metabolism and the high concentration of methane in the anoxic basin, sulfur metabolism pathways in this environment were also examined. In the anoxic basin, the pathways responsible for sulfur metabolism were sulfate activation for sulfonation, sulfate reduction, and hydrogen sulfide biosynthesis (Fig. 6).

The highest abundance of sulfur pathways belonged to sulfate reduction. This pathway made up 64.5% of sulfur metabolism pathways in the LF92 sample. It made up 66% of sulfur metabolism pathways in the UF92 sample and 56.2% of sulfur metabolism pathways in the LF99 sample. Only 8.1% of sulfur metabolism pathways in the sediments belonged to sulfate reduction. Within the sediments, only unclassified organisms had this pathway. In the water column, this pathway was found in unclassified organisms, Deltaproteobacteria, Betaproteobacteria, and Bacteroidetes.

The hydrogen sulfide biosynthesis pathway was only present in the LF99 (6.2%) and sediment samples (33%). This pathway is present in unclassified organisms, Actinobacteria, Betaproteobacteria, and Deltaproteobacteria.

## Discussion

### High cell count and diverse taxa despite harsh conditions

The waters of the anoxic basin support a thriving community, containing 8 × 10^5^–9 × 10^5^ cells/mL. The cell count in the deep waters was higher than similar environments, such as Antarctic subglacial Lake Whillans, which harbors 6.6 × 10^4^–3.7 × 10^5^ cells/mL within the anoxic water^27,28^ as well as the anoxic subglacial water beneath Vatnajökull in Iceland, which harbors 5 × 10^5^ cells/mL^29^. The cell count in the Untersee anoxic basin was also an order of magnitude higher than the anoxic subglacial waters feeding Blood Falls in Antarctica, which harbor 6 × 10^4^ cells/mL^30^, as well as the cell count in Lake Vostok, estimated to be ~ 10^5^ cells/mL^28^. We also compared the anoxic basin to less extreme communities such as lakes in the McMurdo Dry Valleys. A study conducted at Lake Fryxell found that the cell counts in these lakes decreased with depth, with the highest cell counts at approximately 4 × 10^5^ cells/mL^54^. While these values are of the same order of magnitude as the anoxic basin, they are still slightly lower. This suggests the harsh, dark, cold environment of the anoxic basin of Untersee can support diverse life, mirroring the results of our taxonomic analysis. Even though 36% of the community at 92 m, 43% at 99 m, and 45% of the community in the sediment were unclassified, the classified members comprised 52 phyla in total. A 16S rRNA study done on the waters of Lake Fryxell and Lake Miers in the McMurdo Dry Valleys found that the most diverse microbial community was Lake Fryxell, with 49 annotated phyla^55^. This suggests that Lake Untersee is similar in diversity to other Antarctic lakes used as analogs.

Together, the presence of diverse phyla (Fig. 3) along with the relatively high cell counts demonstrates that this analog environment is well suited to support life. If life exists on ocean worlds, it is often envisioned to be low biomass, largely as a result of the dark, cold conditions. Yet, in this analog environment, microbial life is both thriving and diverse.

### Complete and incomplete methanogenesis pathways in Untersee indicate varied sources of energy

The pathway for methanogenesis from CO_2_/H_2_ was detected in the anoxic basin in Euryarchaea and unclassified organisms (with the exception of LF92, where it is also found at low levels in Deltaproteobacteria). There is a high concentration of dissolved methane in this environment, and substantial levels of methane and CO_2_ and H_2_ have been detected in the Enceladus plumes. Methane may play an important role in the metabolic cycle of life that may exist in the sub-ice environments of Enceladus just as it does beneath the ice of Lake Untersee^19^. The low levels of this pathway detected in the Deltaproteobacteria could signify the presence of a partial pathway in these organisms. Euryarchaea are the only known anoxic methanogens, suggesting this pathway is likely to be carried to completion with the production of methane. This finding is consistent with Wand et al. (2006), which posits that methanogenesis in Untersee likely occurs near the sediment and is from a CO_2_/H_2_ source. Hydrogenotrophic methanogenesis also dominates the methane production mechanism in the anoxic sediment of Lake Vanda in the McMurdo Dry Valleys, even though traces of methylotrophic and acetoclastic methanogenesis were present in those sediments, similar to the pathways present in the organisms in Lake Untersee. From this, it is evident using hydrogen as the electron donor for methanogenesis occurs in other extreme environments as well. The escape rates of H_2_ and methane in the plumes of Enceladus cannot be explained purely by abiotic production via serpentinization^32^. Hydrogenotrophic methanogenesis may also be used by potential life on Enceladus, just as it is a source of biotic methane in Lake Untersee.

The pathway for methanogenesis from acetate was present in higher abundance in the genomic DNA than any other methanogenesis pathway. However, although methanogenesis in Untersee is derived mainly from CO_2_ reduction using hydrogen, the pathways we found in Untersee for acetate-based methanogenesis have been reported to be the principal mechanism for methane production in other anoxic sediments such as those of Lake Fryxell^56^. Within the anoxic water column of Lake Untersee, methanogenesis from acetate was present in nearly all major phyla in the water and sediment, including non-methanogenic phyla such as Deltaproteobacteria, Betaproteobacteria, and Firmicutes. Given that these phyla are not known for anoxic methanogenesis, it is likely that they are able to use acetate as their carbon energy source, therefore implying that the compound may be an important source of energy for life in this anoxic environment. Indeed, acetate may well be present within the oceans of Enceladus. We draw this conclusion based on evidence that acetate is the most abundantly present water-soluble organic molecule in carbonaceous chondrites^33^. Silica nanoparticles have been detected in the E-ring of Saturn, which originated from the plumes of Enceladus^34^, and in order to sustain these silica particles within the ring, Enceladus’s core must also contain carbonaceous chondrites^35^. The lack of detection of acetate in the plumes can be explained by the basic pH of Enceladus. In this environment, acetate will most likely be present in the form of an ionized organic (C_2_H_3_O). Ionized organics are water soluble and non-volatile, thus they would evade detection by Cassini’s instrument suite^36^. The presence of abiotic acetate on Enceladus could serve as yet another source of energy in this analog environment*.*

### Other energetic pathways for life

In the microbial communities of the anoxic basin, sulfate reduction pathways were found to dominate sulfur metabolism within the water column^13^. Sulfate is an efficient electron acceptor in anoxic environments and its reduction in anoxic environments can be linked to methane oxidation. This can be seen in other environments such as Lake Fryxell and Lake Vanda in the McMurdo Dry Valleys, where anaerobic methane oxidation is known to take place in the anoxic water columns. This reaction has been found to be coupled with sulfate reduction, allowing organisms to use sulfate as the electron acceptor in the upper and lower anoxic sections^56,57^. The presence of this pathway in the water can facilitate methane oxidation in the water [Equation (1)]^37^.

1 $${\text{CH}}_{{4}} + {\text{ SO}}_{{4}}^{{{2} - }} \to {\text{ HCO}}_{{3}}^{ - } + {\text{ HS}}^{ - } + {\text{ H}}_{{2}} {\text{O}}$$

While compounds detected in the Enceladus plumes indicate the potential for life*,* life with similar terrestrial biochemistry would require organic forms of these compounds in order to use them for metabolic purposes. While traces of HS may have been detected in the plumes, the presence of sulfate within the ocean can fortify the possibility of life. Sulfate can be produced by the interaction of radiolytically produced H_2_ with sulfur ions from the chondritic core. Given that sulfate is soluble in water, it would have escaped detection by Cassini^36^.

Nitrogen compounds are necessary for terrestrial life, and their presence in the plumes of Enceladus could present another potential source of energy. Nitrogen metabolism pathways were present in the samples, with nitrate reduction being the most abundant pathway. It was present in nearly all phyla within the water samples, suggesting that many phyla can use nitrate as an energy source. Nitrate is known to be an efficient electron acceptor in anoxic environments and the reduction of nitrate is a preferred mode of nitrogen metabolism in the absence of oxygen^38^. However, in the anoxic basin of Untersee, nitrate levels were measured at below detectable limits (Table 1). Nitrate may be the limiting compound in this reaction, meaning that nitrate is being used as a nutrient source by the community to the point of depletion. The nitrate reduction pathway could also be an inactive pathway or a potential cryptic pathway that could be activated under specific conditions^39^. One way to answer this question is by studying the metatranscriptomics of the community in which pathways are active.

In addition to acting as efficient electron acceptors, nitrite and nitrate reduction in the community can assist in the anaerobic oxidation of methane. The nitrogen detected in the Enceladus plumes was in organic forms, such as HCN (~ 1%) and NH_3_ (~ 1%), both of which are readily available for microorganisms to use^40^. Nitrite and nitrate could act as anoxic electron acceptors that could not only introduce redox possibility in the oceans of Enceladus, but also allow for more efficient use of the methane as an energy source.

### A potential repository of cells

The anoxic basin of Lake Untersee may act as a potential repository of cells. Near the bottom of the basin and in the sediment, taxa and pathways that were not expected to be present in an anoxic environment were observed. These pathways could belong to organisms that live in the aerobic section but have sunk down to the anoxic basin and sediment. While most classified organisms were members of taxonomic groups that are known to survive in anoxic environments, such as Methanomicrobia and Desulfurobacterales, we also identified Cyanobacteria, a phylum known to use both oxygen and light to survive, in the water column (< 1%). The presence of a small number of Cyanobacteria (~ 1%) within the sediment suggests the sediment too may be harbor a record of cellular life from the overlying lake. Cyanobacterial mats are also known to be present in Lake Untersee’s shallower, oxygenated waters.

In addition, the superoxide radical degradation pathway was identified in the sediment. This pathway prevents oxygen toxicity from oxygen produced from metabolic processes in organisms which live in aerobic environments^41^. In the anoxic zone, there is no evidence of oxygen production, and therefore this pathway is unlikely to have evolved in organisms that live there. We hypothesize that this pathway isn’t active at depth, something future metatranscriptomic analysis of the sediment could help to clarify.

The presence of a deep cell repository could have a range of important implications. It could provide additional sources of sustenance for organisms that live in the anoxic zone, and it could play an important role in the nutrient cycling of the lake. It may also suggest that the sediment–water interface on Enceladus could be a prime place to look for records of not only active but also past life.

## Conclusion

Despite the harsh environmental conditions in the anoxic waters of Lake Untersee, the many metabolic pathways and chemical sources of energy have led to a taxonomically and metabolically diverse community. The high cell counts and diversity of microbial life in the anoxic basin also demonstrate that the environment is highly habitable, suggesting that methane-rich ocean worlds like Enceladus may be capable of maintaining diverse and thriving ecosystems despite the cold, dark conditions. Life may have originated independently within the sub-ice ocean of Enceladus—studies of the adaptive strategies used by the metabolically diverse microorganisms within the anoxic water and sediment of Lake Untersee provide additional confidence that such an ecosystem, using a similar suite of metabolically important compounds as identified in the plumes of Enceladus, could be sustained within the depths of that distant icy ocean world.

Lake Untersee’s ecosystem provides many opportunities for future work. The presence of a large percentage of unclassified organisms in the community, also known as microbial dark matter, is in part due to the limitations of our understanding of the evolution and physiology of organisms in extreme environments^42^. Ice-covered Antarctic lakes have been little studied using modern molecular techniques, leading to a paucity of identifications in our databases; by using Metagenome Assembled Genomes (MAGs) obtained from the assemblies, the representation of organisms from extreme environments in current databases can be increased^43^.

With regard to life detection on ocean worlds, future work should focus on developing exploration and life-detection strategies for Enceladus. While nucleobases are common in space^58^, XNA sequencing is not yet on the horizon for near-term space missions, and life on Enceladus of course may be based on different biochemistry than life on Earth. Nevertheless, genomic studies of analog environments offer insights into the byproducts and other chemical biosignatures that life may leave behind in anoxic conditions, thereby helping to hone life detection targets and strategies for ocean worlds.

## Methods

### Sample collection

Water (1L from each depth) and sediment samples were collected at Lake Untersee in the Fall of 2018. Samples were collected from a hole drilled using Jiffy drills (Feldmann Engineering) with 20-cm diameter bits to provide access to the water column below the thick lake ice of the anoxic basin (S71.3556°1, E013.42493°). Water samples were taken with a 2.5L, model 1010 Niskin Water Sampler (General Oceanics, Miami, FL) which was cleaned with 95% ethanol (Sigma-Alrdrich, Munich, Germany) and RNase Away (Thermofisher, Waltham, MA) prior to being lowered into the water. Water was collected from depths of 75 m—the oxycline—as well as 92 m and 99 m in the stratified anoxic section of the column. Water was collected from the shallower depths first (75 m, 92 m, and 99 m in order) to avoid disturbing the water column. Anoxic sediments at 100 m were collected using a stainless steel Ekman dredge sampler (6 × 6 × 6 inches, Wildco, Yulee, FL) targeting the upper top 10 cm of sediment. The sediment sample was collected from the same hole five days after the water collection. The dredge was cleaned with ethanol and RNase Away.

### Sample preparation

After collection, the sediment samples were transferred to cryotubes using sterile sampling tools, then placed in a liquid nitrogen primed MVE cryoshipper (LabRepCo, Horsham, PA) at − 180 °C. Before being placed in the cryoshipper, water samples were filtered using an InnovaPrep Concentrating Pipette (InnovaPrep, Drexel, MO). The turbid nature of the water made it impossible to work with only one filter size. The particulates present would have clogged a small filter almost immediately, while using only a larger filter would have resulted in losing many of the organisms present. In order to avoid these problems, the water was filtered twice through a large and a small filter. The samples were initially filtered through a 0.45 µm hollow fiber filter in order to collect any particulates and larger cells (designated as LF for “Large Fraction”). The filtrate was then passed through a 0.05 µm hollow fiber filter in order to collect cells using the smallest available filters for the InnovaPrep Concentrating Pipette (designated as UF for “Ultrafine Fraction”). The filtered cells were diluted in Tris elution buffer. The filtrate was then pipetted into cryovials and transferred to the cryoshipper. The cryovial samples were transported in the primed cryoshipper back to Georgetown University where they were stored in a − 80 °C freezer.

Non-filtered water samples were collected in 1L wide-mouth amber Nalgene bottles. They were stored in coolers placed in open Antarctic weather conditions, which did not exceed 0 °C during the expedition. They were stored at − 20 °C in Cape Town for 20 days before being shipped to Georgetown University on dry ice. Upon arrival, they were stored in a − 80 °C freezer.

### Cell counts

Cell counts were carried out on non-filtered water at Georgetown’s Flow Cytometry and Cell Sorting Shared Resource Center. In order to separate the cells from particulates, SYTO 40 fluorescent nucleic acid stain was used to stain the cells and count them using the flow cytometer (BD FACSAria IIu Cell Sorter with laser set at 400 nm, FCSExpress 7 software used). In order to count the absolute number of cells, a comparison with Trucount beads (BD Biosciences, San Jose, CA), which have a predetermined number of fluorescent beads, was done. Cell counts were carried out for all water samples, 75 m, 92 m and 99 m (Table 1). The SYTO 40 dye does not differentiate between living and dead cells, and the values obtained reflect the total number of cells present in the samples. Because inefficient cell detachment and separation from matrix particles complicate results from flow cytometry, we did not collect cell counts for the sediment sample.

### Nutrient analysis

Duplicate 10 mL non-filtered samples were placed in acid-washed bottles and sent to Woods Hole Oceanic Institute Nutrient Analytical Facility in Woods Hole, MA. Samples were analyzed on a four-channel segmented flow AA3 HR Autoanalyzer to determine dissolved nutrient concentration in aquatic ecosystems, specifically nitrate + nitrite, ammonium, phosphate, and silicate (Table 1).

### Extraction

All extractions were carried out in an AirClean Systems ISO 5 laminar flow hood at Georgetown University. DNA from the sediment was extracted in duplicate. To lyse the cells, 500 mg of sample was added to 500 µL of phenol–chloroform-isopropanol solution at a 25:24:1 concentration (Sigma-Alrdrich, Munich, Germany) in Lysis Matrix E tubes (MPBio, Santa Ana, CA) subjected to high velocity bead-beating with a FastPrep 24 5G (Qiagen, Inc., Valencia, CA) at 5.5 m/s for 30 s. The genomic material was then separated from the organics in the sample by rapid centrifugation of the lysate at 4 ˚C, at a speed of 16,000 g for 5 min along with 500µL chloroform-isopropanol alcohol in a phase lock heavy gel tube. The genomic material (phase separated from the organics) was moved to a clean tube and left to incubate at room temperature for an hour. After this preparation step, a Qiagen AllPrep DNA/RNA Extraction Kit (Qiagen Inc., Valencia, CA) was used to purify the DNA out of the sample alongside sample blanks to track contamination.

For the cells concentrated out of the anoxic sample water—the focus of this study—ultrafine (0.05–0.45 µm) and large fractions (> 0.45 μm) concentrated from both 92 m and 99 m depths were extracted in duplicate, for a total of four extractions. 250 µL of cells concentrated in Elution Buffer (InnovaPrep, Drexel, MO) along with 500µL of 25:24:1 phenol–chloroform-isopropanol bead-beated at 5.5 m/s for 30 s in Lysis Matrix E tube followed by a Qiagen AllPrep DNA/RNA extraction kit was used to extract and purify DNA from the cells. Sample blanks were again used to ensure no contamination.

The yield from the samples was measured using the high sensitivity dsDNA Qubit Assay kit (Thermofisher, Waltham, MA). All replicate extractions were measured before sequencing to ensure the samples had enough DNA.

### Sequencing and data analysis

The DNA extractions from the sediment were sequenced on the Illumina MiSeq platform at 300 bp paired-end MiSeq reads sequenced at the Georgetown University Genomics and Epigenomics Shared Resource Center.

DNA extractions from the water samples, which were generally low-yield, were prepared with the Accel-NGS® 1S Plus DNA Library Kit (Swift Biosciences, Ann Arbor, MI). The sequencing was then completed using the paired-end 250 bp Illumina NextSeq platform. The samples were sequenced at the University of Illinois at Chicago Genome Research Core. Due to constraints in laboratory services during the COVID-19 pandemic, we were unable to send this final sample to the same facility. Although both sequencing platforms used Illumina technology, it should be noted that the difference in the sequencing platform used for the water samples versus the sediment sample may bias the results, and this should be considered when interpreting the data.

The raw reads were trimmed and the reads above Q30 threshold were selected using Trimmomatic using default settings^44^. The quality of the reads was checked with FastQC^45^. Details about the number of reads, number of ORFs, and read lengths are presented in Supplementary Tables 1, 2 and 3. The assemblies were built with the MEGAHIT pipeline with default settings^46^.

MetaPathways V2.5^24^ was used to annotate the assemblies using several databases including RefSeq^26^, KEGG^47^, and MetaCyc^48^. The samples were normalized using RPKM (Reads Per Kilobase per Million mapped reads)^24^.

While both read and assembly-based methods were used, in order to have high quality alignment and functional annotation^20^, we used metagenomic assemblies for our downstream analyses. More details can be found in the Supplementary Information.

#### Taxonomic profiles

The taxonomic profile of the community was made using several different software tools including, Kraken2^23^, Kaiju^22^, and Lowest Common Ancestor Star algorithm (LCA*)^21^, a method that assigns taxonomy to contigs instead of open reading frames using the least common ancestor algorithm and voting theory method. The Lowest Common Ancestor Star method cannot assign a deep taxonomic classification to housekeeping genes since they are ubiquitous in organisms.

#### Functional analysis

Where possible we linked the function to taxonomy at the contig level using LCA*. The quality of the UF99 sample is poorer than that of other assemblies; there are fewer reads mapped in this assembly (Table S2), thus fewer ORFs were found and annotated in this sample.

Functional annotation and pathway prediction were done using MetaPathways V2.5^24^, a modular pipeline for open reading frame (ORF) prediction, functional and taxonomic annotation using the RefSeq database, ORF count normalization (for both sequencing depth and ORF length), and the creation of environmental pathway genome databases (ePGDBs) based on a well-curated database of metabolic pathways and components representing all domains of life^24,25,49^. ORF counts were normalized using Reads Per Kilobase per Million mapped reads (RPKM). Still, it is important to note that given different cell counts, read depth and coverage vary among samples. Here focus was placed on pathways responsible for nitrogen, sulfur and methane metabolism and detox pathways. Data was visualized in R 3.5.3^50^, with ggplot2^51^.

## Supplementary Information

Supplementary Information.

## Data Availability

All genomic data is available from the NCBI Sequence Read Archive under BioProject #PRJNA783029 (https://www.ncbi.nlm.nih.gov/bioproject/?term=PRJNA783029).
