# Author Correction: Survival strategies of an anoxic microbial ecosystem in Lake Untersee, a potential analog for Enceladus

**DOI:** 10.1038/s41598-022-22009-2

**Published:** 2022-10-11

**Authors:** Nicole Yasmin Wagner, Dale T. Andersen, Aria S. Hahn, Sarah Stewart Johnson

**Affiliations:** 1 Department of Biology, Georgetown University, 3700 O Street NW, Washington, DC 20057 USA; 2 SETI Institute, Mountain View, CA USA; 3 Koonkie Inc, Menlo Park, CA USA; 4 Science, Technology, and International Affairs Program, Georgetown University, Washington, DC USA

Correction to: *Scientific Reports* https://doi.org/10.1038/s41598-022-10876-8, published online 05 May 2022

The Data Availability section in the original version of this Article was omitted.

It now appears as below:

**Data availability**

All genomic data is available from the NCBI Sequence Read Archive under BioProject #PRJNA783029 (https://www.ncbi.nlm.nih.gov/bioproject/?term=PRJNA783029).

The original Article has been corrected.

