# Supplementary material for: Survival strategies of an anoxic microbial ecosystem in Lake Untersee, a potential analog for Enceladus
Source: Sci Rep. 2022 May 5;12:7376. doi: 10.1038/s41598-022-10876-8 (PMC9070616; doi:10.1038/s41598-022-10876-8)
Supplement: Supplementary file 1 — Supplementary Information. [file 41598_2022_10876_MOESM1_ESM.docx]

**Supplementary Information**

**Fig. S1 | The breakdown of the classified and unclassified taxonomic communities.** A. Indicates the percentage of classified and unclassified organisms in the community. B. Shows the percentage of phyla present that make up more than 1% of the community. Different classes belonging to Proteobacteria are shown in different shades of red.


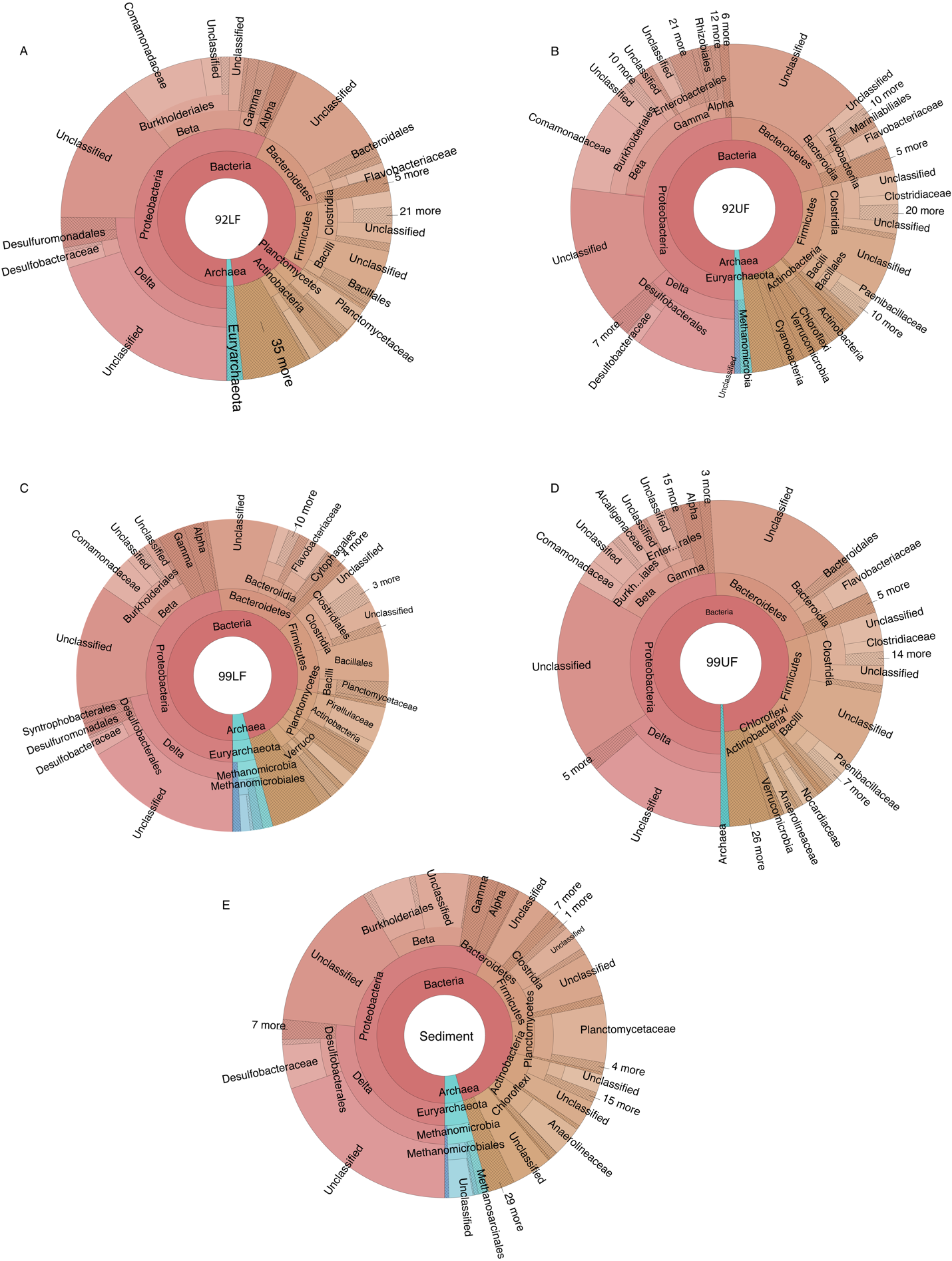


**Fig. S2 | Krona plots of the taxonomic profile of classified communities down to the order level.**  A. Classified taxonomy for the large filter community at 92m (LF92). B. Classified taxonomy for the ultrafine filter community at 92m (UF92). C. Classified taxonomy for the large filter community at 99m (LF99). D. Classified taxonomy for the ultrafine filter community at 99m (UF99). E. Classified taxonomy for the sediment.

**Table S1** **|** Read information.

| Composite stats for all files | Number of lines in SAM file | Number of unmapped lines | Number of mapped lines | Number of singletons | Number of read 1 | Number of read 2 | Number of multireads | Number of secondary hits | Number distinct reads mapped | Number distinct reads unmapped |
| --- | --- | --- | --- | --- | --- | --- | --- | --- | --- | --- |
| LF92 | 37352359 | 4526965 | 32825394 | 2492819 | 18678810 | 18673549 | 163699 | 167585 | 32657809 | 4526965 |
| LF99 | 22136983 | 3500000 | 18636983 | 1873103 | 11071451 | 11065532 | 117559 | 120309 | 18516674 | 3500000 |
| XS18 | 54836297 | 11247580 | 43588717 | 5868213 | 27621254 | 27215043 | 1555021 | 1618611 | 41970106 | 11247580 |
| UF92 | 26945341 | 3467366 | 23477975 | 1887475 | 13476895 | 13468446 | 155855 | 159867 | 23318108 | 3467366 |
| UF99 | 14806109 | 8155582 | 6650527 | 4210027 | 7404858 | 7401251 | 38816 | 39563 | 6610964 | 8155582 |

**Table S2 |** ORF information.

| Mapping stats for the sample | Number of Contigs | Number of ORFs in sample | Number of ORFs in pathway table | Total contig cover length | Total Contig Length | Total Genome Length | Percentage contig coverage | Total ORF Length | Total num of mappable reads | Total num of unmappable reads | Number of total reads | Percentage of mapped reads | Number of multireads | Percentage of multireads | Avg rpkm across ORFs in sample | Avg rpkm across ORFs pwy table |
| --- | --- | --- | --- | --- | --- | --- | --- | --- | --- | --- | --- | --- | --- | --- | --- | --- |
| LF92 | 404664 | 632053 | 0 | 344657059 | 345247438 | 345247438 | 99.83% | 302607353 | 32657809 | 4526965 | 37184774 | 87.83% | 163699 | 0.44% | 2.38 | 0 |
| LF99 | 328450 | 497885 | 0 | 262104760 | 262525548 | 262525548 | 99.84% | 229826926 | 18516674 | 3500000 | 22016674 | 84.10% | 117559 | 0.53% | 3.09 | 0 |
| XS18 | 42105 | 214981 | 0 | 204607098 | 204638628 | 204638628 | 99.98% | 181492070 | 41970106 | 11247580 | 53217686 | 78.86% | 1555021 | 2.92% | 5.19 | 0 |
| UF92 | 283932 | 433691 | 0 | 217425121 | 218080921 | 218080921 | 99.70% | 191992780 | 23318108 | 3467366 | 26785474 | 87.06% | 155855 | 0.58% | 4.18 | 0 |
| UF99 | 8143 | 45472 | 0 | 38920829 | 38934018 | 38934018 | 99.97% | 34623227 | 6610964 | 8155582 | 14766546 | 44.77% | 38816 | 0.26% | 15.78 | 0 |

**Table S3 |** Sequence quality information.

|  | Number of sequences | Number of sequences shorter than minimum length of sequences | Average length of sequences | Minimum length of sequences | Maximum length of sequences |
| --- | --- | --- | --- | --- | --- |
| LF92-Before | 404664 | 404664 | 853.1706255 | 200 | 190157 |
| LF92-After | 404664 | 0 | 853.1706255 | 200 | 190157 |
| LF99-Before | 328450 | 328450 | 799.2861866 | 200 | 177294 |
| LF99-After | 328450 | 0 | 799.2861866 | 200 | 177294 |
| XS18-Before | 1352288 | 1352288 | 670.3569839 | 200 | 158057 |
| XS18-After | 42105 | 0 | 4860.197791 | 2000 | 158057 |
| UF92-Before | 283932 | 283932 | 768.0744721 | 200 | 252555 |
| UF92-After | 283932 | 0 | 768.0744721 | 200 | 252555 |
| UF99-Before | 206037 | 206037 | 736.9625941 | 200 | 190157 |
| UF99-After | 8143 | 0 | 4781.286749 | 2000 | 190157 |

**Table S4** | Lake Untersee Anoxic Basin conductivity, pH and DO Measurements.

| depth (m) | conductivity (µS/cm) | pH | DO (mg/l) | T (˚C) |
| --- | --- | --- | --- | --- |
| 4.98 | 500 | 10.73 | 18.46 | 0 |
| 9.99 | 505 | 11.03 | 20.55 | 0 |
| 15 | 500 | 11.01 | 20.9 | -0.05 |
| 20 | 504 | 10.99 | 19.91 | 0.02 |
| 25 | 505 | 10.89 | 19.47 | 0 |
| 29.99 | 504 | 10.94 | 19.11 | 0.05 |
| 35 | 504 | 10.83 | 18.91 | 0.04 |
| 40 | 503 | 10.67 | 18.7 | 0.26 |
| 45 | 504 | 10.58 | 18.68 | 0.29 |
| 50.03 | 516 | 10.42 | 18.54 | 3.96 |
| 55 | 518 | 10.35 | 18.53 | 4.01 |
| 60 | 518 | 10.31 | 18.5 | 4.01 |
| 65.03 | 515 | 10.03 | 16.65 | 4.24 |
| 70 | 540 | 8.97 | 3.9 | 4.85 |
| 72 | 544 | 8.4 | 0.26 | 4.94 |
| 73 | 545 | 8.13 | 0.03 | 4.94 |
| 74 | 544 | 7.87 | 0.01 | 4.93 |
| 76 | 546 | 7.75 | 0.01 | 4.91 |
| 77 | 546 | 7.64 | 0.01 | 4.91 |
| 80 | 563 | 7.04 | 0 | 4.97 |
| 81 | 593 | 6.72 | 0.02 | 4.95 |
| 83 | 669 | 6.47 | 0.01 | 4.83 |
| 85 | 769 | 6.33 | 0.01 | 4.69 |
| 87 | 927 | 6.23 | 0.02 | 4.48 |
| 88.02 | 991 | 6.2 | 0.01 | 4.39 |
| 89 | 1035 | 6.17 | 0.02 | 4.32 |
| 91 | 1152 | 6.12 | 0.01 | 4.15 |
| 92 | 1181 | 6.12 | 0.02 | 4.09 |
| 93 | 1227 | 6.11 | 0.02 | 4 |
| 94 | 1260 | 6.1 | 0.01 | 3.92 |
| 95 | 1275 | 6.1 | 0.02 | 3.87 |
